# Enzymatic Activities and DNA Substrate Specificity of *Mycobacterium tuberculosis* DNA Helicase XPB

**DOI:** 10.1371/journal.pone.0036960

**Published:** 2012-05-16

**Authors:** Seetha V. Balasingham, Ephrem Debebe Zegeye, Håvard Homberset, Marie L. Rossi, Jon K. Laerdahl, Vilhelm A. Bohr, Tone Tønjum

**Affiliations:** 1 Centre for Molecular Biology and Neuroscience (CMBN) and Department of Microbiology, University of Oslo, Oslo, Norway; 2 Department of Microbiology, Oslo University Hospital Rikshospitalet, Oslo, Norway; 3 Laboratory of Molecular Gerontology, NIH Biomedical Research Center, National Institute on Aging, National Institutes of Health, Baltimore, Maryland, United States of America; 4 Bioinformatics Core Facility, Department of Informatics, University of Oslo, Oslo, Norway; Saint Louis University, United States of America

## Abstract

XPB, also known as ERCC3 and RAD25, is a 3′→5′ DNA repair helicase belonging to the superfamily 2 of helicases. XPB is an essential core subunit of the eukaryotic basal transcription factor complex TFIIH. It has two well-established functions: in the context of damaged DNA, XPB facilitates nucleotide excision repair by unwinding double stranded DNA (dsDNA) surrounding a DNA lesion; while in the context of actively transcribing genes, XPB facilitates initiation of RNA polymerase II transcription at gene promoters. Human and other eukaryotic XPB homologs are relatively well characterized compared to conserved homologs found in mycobacteria and archaea. However, more insight into the function of bacterial helicases is central to understanding the mechanism of DNA metabolism and pathogenesis in general. Here, we characterized Mycobacterium tuberculosis XPB (Mtb XPB), a 3′→5′ DNA helicase with DNA-dependent ATPase activity. Mtb XPB efficiently catalyzed DNA unwinding in the presence of significant excess of enzyme. The unwinding activity was fueled by ATP or dATP in the presence of Mg^2+^/Mn^2+^. Consistent with the 3′→5′ polarity of this bacterial XPB helicase, the enzyme required a DNA substrate with a 3′ overhang of 15 nucleotides or more. Although Mtb XPB efficiently unwound DNA model substrates with a 3′ DNA tail, it was not active on substrates containing a 3′ RNA tail. We also found that Mtb XPB efficiently catalyzed ATP-independent annealing of complementary DNA strands. These observations significantly enhance our understanding of the biological roles of Mtb XPB.

## Introduction

*Mycobacterium tuberculosis* is a human pathogen that targets macrophages and causes significant mortality and morbidity especially in the developing countries. Notably, *M. tuberculosis* is resistant to reactive oxygen intermediates (ROI) and reactive nitrogen intermediates (RNI), which are produced and secreted by host macrophages specifically to attenuate the deleterious effects of intracellular pathogens [1]. *M. tuberculosis* also survives environmental stresses such as exposure to UV irradiation, nutrient starvation, dehydration and low temperature during host transfer [2], in addition to endogenous stresses such as low pH and hypoxia. This suggests that *M. tuberculosis* has efficient DNA repair and/or other mechanisms that protect against ROI- and RNI-induced cellular damage. Consistent with this notion, *M. tuberculosis* expresses many well conserved genes that play roles in base excision repair (BER), nucleotide excision repair (NER), recombination repair and the SOS response to cellular stress [3], [4]. However, homologs of mismatch repair (MMR) genes, such as *mutL*, *mutS* and *mutH*, have not been identified in *M. tuberculosis* [4], [5].

Helicases are ubiquitous enzymes that play essential roles in DNA repair, recombination, replication, transcription and RNA processing [6], [7]. DNA damage recognition requires an initial step by helicases, identifying helical distortions in the DNA. Typically, helicases move along the phosphodiester backbone of duplex nucleic acid strands in a directional manner, using energy derived from NTP/dNTP hydrolysis to unwind and separate the complementary nucleic acid strands. DNA helicases are thus among the first proteins that encounter DNA damage and play important roles in its repair.

The XPB/ERCC3/RAD25 protein, belonging to the helicase superfamily 2 (SF2), is an integral subunit of the eukaryotic basal transcription factor IIH (TFIIH), which is involved in proofreading of transcription initiation and NER [8], [9]. The TFIIH complex comprises up to 10 protein subunits and contains two helicases: XPB and XPD. XPB is missing in the genomes of many prokaryotes, but several actinobacterial species, including *M. leprae, M. tuberculosis* and *Kineococcus radiotolerans* express a XPB homolog [10], [11]. In contrast, the TFIIH XPD/ERCC2 homolog DinG, (damage-inducible G) is commonly expressed in many bacterial species [12]. The function of these two helicases, XPB and DinG, in bacteria, is not well understood. Homologs of other subunits of the TFIIH complex, such as p44 and p52, have not been found in bacteria. The p44 and p52 subunits are believed to have crucial roles in regulating XPB and XPD enzymatic function in mammals [13]. In humans, mutations in *XPB* or *XPD* cause defects in both RNA transcription and DNA repair, leading to at least three severe genetic disorders: Xeroderma pigmentosum (XP), Cockayne syndrome (CS) and Tricothiodystrophy (TTD) [14], [15], [16]. The biological consequences of defects in bacterial XPB or DinG are still poorly understood.

NER recognizes and repairs bulky DNA adducts and helix distorting lesions, including photoproducts [17]. In *Escherichia coli* and mycobacteria, NER requires the UvrABC excinuclease enzyme complex and the UvrD helicase [18], [19], [20], while in human cells, NER is a complex pathway involving many proteins in addition to the TFIIH complex [21], [22]. Mycobacteria have two UvrD helicase homologs: UvrD1 and UvrD2 [23], [24], [25].

Eukaryotic XPB contains a central helicase core domain and N- and C-terminal domains [8]. Bacterial XPB also contains the main helicase core and a shorter N-terminal domain, but lacks the C-terminal domain [10], [11]. The N-terminal domain of eukaryotic XPB interacts with the p52 subunit of TFIIH which stimulates the ATPase activity of XPB [13]. The three-dimensional crystal structure of a XPB homolog from *Archaeoglobus fulgidus* revealed the presence of two RecA-like helicase domains and a short N-terminal domain which showed structural similarity to the mismatch recognition domain of MutS, termed the damage recognition domain (DRD) [26]. This protein structure also revealed two sequence motifs that may be functionally important in XPB, a long flexible loop unique to XPB (RED) and a Thumb domain (ThM) [26].

*M. tuberculosis* XPB is an ATP-dependent 3′→5′ DNA helicase, whose ATPase activity is preferentially stimulated by single stranded DNA (ssDNA) [10]. However, the biological role of XPB in bacteria, including *M. tuberculosis* and in archaea, is at present poorly understood. Furthermore, Mtb XPB contains key domains that can help to understand the human XPB (hXPB). To improve our understanding of its biological role, we have purified and characterized recombinant Mtb XPB, focusing on its catalytic helicase activities, extended DNA substrate specificities and strand annealing activities. Here, we report the novel observation that Mtb XPB exhibited ATP-independent strand annealing of complementary DNA strands, in addition to its DNA unwinding activity. These observations elucidates the important role of this helicase in genome maintenance and pathogenesis of *M. tuberculosis*.

## Results

### Structural and sequence analysis of Mtb XPB and homologs

**Figure 1:**
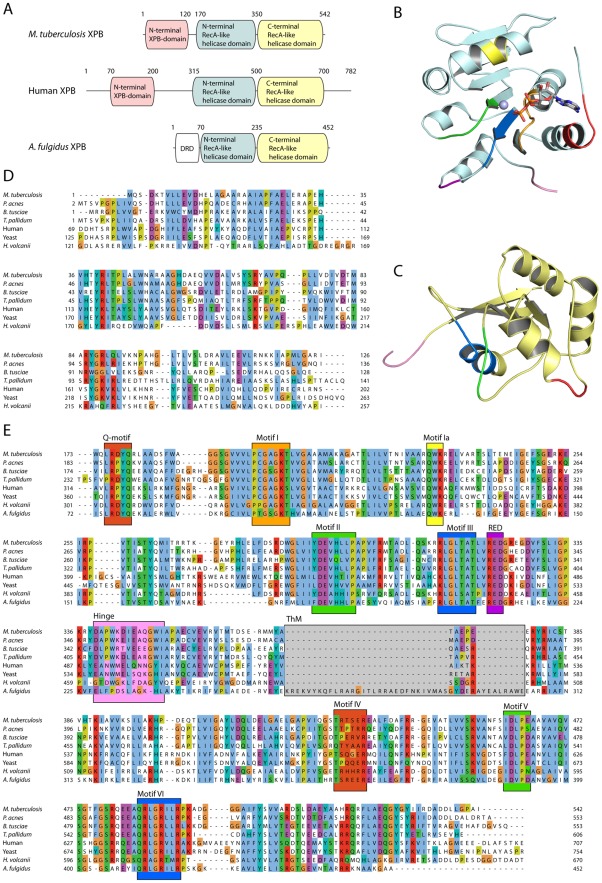
XPB domain organization and functional motifs. A) Domain organization showing the N- and C-terminal RecA-like helicase domains and the N-terminal domain unique to the XPB family of proteins. This latter domain is found in eukaryote, bacterial and some archaeal XPBs, but appears to be replaced by a shorter DNA damage recognition domain (DRD, see [26]) in *A. fulgidus* and a subset of archaea. Numbers indicate the approximate domain boundaries. B) Structural model of the Mtb XPB N-terminal RecA-like helicase domain (in light cyan) showing bound ADP (sticks) and a divalent cation (sphere), the Q-motif (colored red) [31], the RED motif (purple) conserved in the XPB family [26] and the helicase motifs I (orange), Ia (yellow), II (green) and III (blue). The hinge region linking the two RecA-like domains is shown in pink. C) Model of the Mtb XPB C-terminal RecA-like helicase domain (in yellow) with helicase motifs IV (red), V (green) and VI (blue). The hinge region is shown in pink. D) A multiple sequence alignment (MSA) of XPB homologs demonstrates that the N-terminal domain unique to the XPB family of proteins is present in eukaryotes, in yeast (*Saccharomyces cerevisiae* RAD25, NCBI Refseq identifier NP_012123) and human XPB/ERCC3 (NP_000113), in bacteria, *e.g. M. tuberculosis* H37Rv (NP_215376), *Propionibacterium acnes* (YP_003580697), *Bacillus tusciae* (YP_003589555) and *Treponema pallidum* (NP_218820) and in some archaea, including *Haloferax volcanii* (YP_003535766). Sequence numbering is given at the line ends. E) An MSA of the two RecA-like helicase domains of XPB from the three domains of life shows the location of classical helicase motifs, the hinge region linking the two domains, the Q- and RED-motifs and the flexible thumb motif (ThM, grey) that is unique to a subset of archaea including *A. fulgidus* (NP_069194). The coloring of the motifs is identical to panels B) and C).

Sequence searching in publicly available databases revealed prokaryotic homologs of eukaryotic XPB in the majority of archaea, in most spirochaetes and actinobacteria, including the mycobacteria, in some firmicutes, but in few, if any, proteobacteria, cyanobacteria, chlamydiae, or bacteroidetes. Within the XPB protein family, the two core RecA-like helicase domains (Fig. 1 A–C), found in all SF2 helicases/translocases, were highly conserved (Fig. 1E). In addition, there was a conserved N-terminal domain (Fig. 1 A and D) of 120–130 residues that might be unique to XPB. This latter domain appeared to be present in all the bacterial and eukaryotic XPB homologs and also in the family Halobacteriaceae of archaea. In most archaea, however, including *A. fulgidus*, this domain had been replaced with a shorter domain termed the DNA damage recognition domain (DRD, Fig. 1A) by Fan *et al.* [26]. Secondary structure predictions and sequence comparisons suggested that the archaeal DRD and the N-terminal domain of bacterial and eukaryotic XPBs are structurally unrelated.

Protein structure disorder predictions indicated that the flexible N-terminus (50–70 residues in human XPB), the C-terminal ∼80 residues and the linker between the N-terminal and the helicase domains (approximately residues 200–260) were structurally disordered in human and other vertebrate XPB homologs and that none of these disordered segments were present in Mtb XPB. The 3D structure of the N-terminal XPB-domain could not be reliably modeled, but secondary structure predictions suggested that the domain had a mixed α/β-class structure. Some residues and short segments of the N-terminal domain appeared to be almost universally conserved in XPB in bacteria/eukaryotes, including Mtb XPB. 3D models of the N- and C-terminal helicase domains of Mtb XPB were generated with comparative modeling based on the experimentally determined structure of *A. fulgidus* XPB [26]. The classical helicase motifs [27] are shown mapped onto the structural models (Fig. 1, B and C) and in the multiple sequence alignments of bacterial, archaeal and eukaryotic XPBs (Fig. 1E).

### Nucleic acid binding and unwinding activity of Mtb XPB

**Figure 2:**
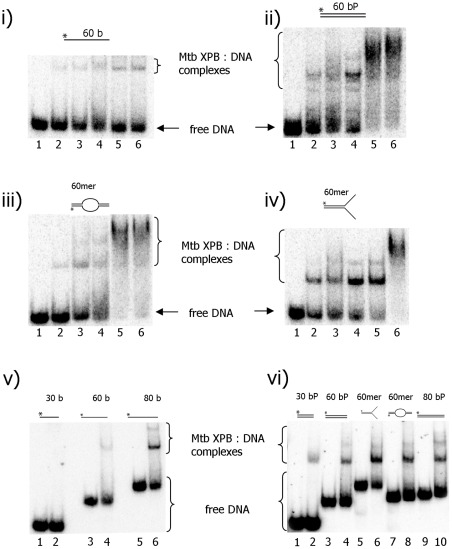
Mtb XPB binds DNA substrates. Representative gels of resolved Mtb XPB-DNA binding mixtures using the DNA substrates given in Table S1. Increasing Mtb XPB concentrations were incubated with the indicated DNA substrate (100 pM) for 15 min on ice in EMSA buffer. i) ssDNA (T1), ii) duplex DNA (T1+B2), iii) bubble DNA ( T1+B3), iv) forked DNA (T1+B1). Lane 1. no enzyme; lanes 2–6. Mtb XPB [nM] 500, 1000, 2000, 4000 and 5000, respectively. v) 2000 nM Mtb XPB was incubated with ssDNA substrates containing 30 (B5, 1 & 2); 60 (T1, 3 & 4) and 80 (C80, 5 & 6) bases. Lanes 1, 3 and 5–reactions without enzyme; lanes 2, 4 and 6–reactions with enzyme. vi) 2000 nM Mtb XPB was incubated with 30 bp duplex (B5+B6, l & 2), 60 bp duplex (T1+B2, 3 & 4), forked DNA (T1+B1, 5 & 6), bubble DNA (T1+B3, 7 & 8) and 80 bp duplex (C80+G80, 9 & 10). Lanes 1, 3, 5, 7 and 9- reactions without enzyme; lanes 2, 4, 6, 8 and 10–reactions with enzyme.

In order to understand the role of helicase XPB in DNA repair in *M. tuberculosis*, the DNA substrate requirements and specificity of this helicase were investigated using a variety of model DNA substrates. Initially, the DNA binding activity was examined in the presence of increasing concentrations of enzymes and 100 pM DNA substrates using an electrophoretic mobility shift assay (EMSA). As shown in Fig. 2 i–iv, Mtb XPB bound to all the DNA substrates examined. However, it bound more efficiently to the duplex DNA, bubble and forked DNA structures than to ssDNA (Fig. 2 ii–iv, respectively). Binding affinity of Mtb XPB increased with increasing length of the ssDNA (Fig. 2 v). A weak binding to a 30 bp duplex was seen, however, a 30 base ssDNA substrate did not bind to Mtb XPB (Fig. 2 v and iv, lane 2). The binding affinity to a forked DNA substrate appeared to be stronger than to the other substrates tested (Fig. 2 iv and vi). While the Mtb XPB bound duplex DNA and bubble DNA, the enzyme was unable to exert its unwinding activity on these substrates (data not shown).

**Figure 3:**
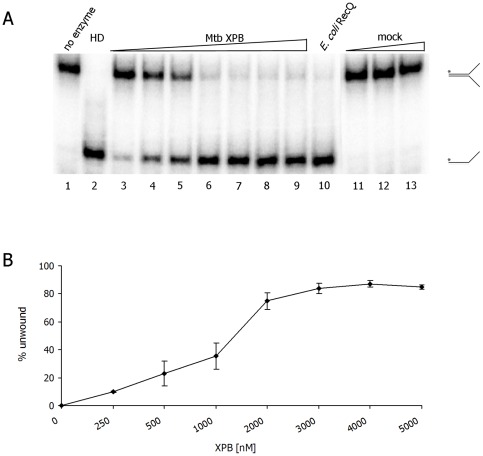
Titration of Mtb XPB unwinding activity. DNA unwinding activity was titrated in the presence of 1 nM forked DNA (T1+B1- a 30mer complementary region with non-complementary 30mer tails) and increasing concentrations of Mtb XPB. A) Representative gel analysis showing unwinding reaction products. Lanes: 1. no enzyme; 2. HD-heat-denatured substrate; 3. 250 nM; 4. 500 nM; 5. 1000 nM; 6. 2000 nM; 7. 3000 nM; 8. 4000 nM; 9. 5000 nM; 10. *E. coli* RecQ (10 nM); 11–13. mock dilutions, 1∶1, 1∶10 and 1∶100, respectively. B) Quantitation of unwinding activity of Mtb XPB. The average of 3 independent experiments and standard deviations (error bars) are shown.

Next, the linear range of the DNA unwinding reaction was determined by incubating a forked DNA substrate with increasing concentrations of Mtb XPB (Fig. 3). Mtb XPB unwound <10% of the input DNA when present at 250 nM, but unwound nearly 80% of the DNA substrate when present at ≥2 µM. In contrast, *E. coli* RecQ, which was used as the positive control, unwound the same substrate to nearly 100% at a 10 nM concentration under the same experimental conditions (Fig. 3). Thus, efficient Mtb XPB unwinding activity was observed only under these *in vitro* conditions and when the enzyme was present in relatively high concentration. This finding is similar to previous reports showing that eukaryotic XPB helicases possess weak unwinding activity *in vitro* [28]. To investigate whether contaminating host proteins were responsible for the DNA unwinding attributed to recombinant Mtb XPB, we performed helicase assays in which the DNA substrate was incubated with volume titration of ‘mock’ purification from *E. coli* host strain harboring vector without Mtb *XPB* insert. These showed no unwinding activity (Fig. 3, lanes 11–13).

### Metal ion and nucleotide requirements for Mtb XPB unwinding activity

**Figure 4:**
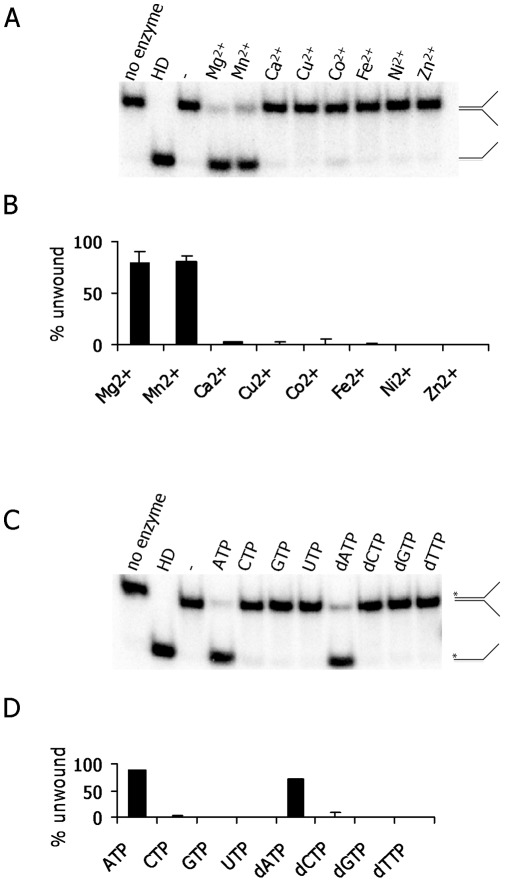
Influence of divalent cations and nucleotide cofactors on Mtb XPB DNA unwinding activity. A) Representative gel showing products of DNA unwinding. Labeled forked DNA substrate (T1+B1- a 30mer complementary region with non-complementary 30mer tails) was incubated with 2000 nM Mtb XPB in the presence of 2 mM ATP and 2 mM of each metal ion for 30 min at 37°C. HD – heat-denatured DNA substrate. The control reaction lacked metal ion (-). B) Quantitation of Mtb XPB unwinding activity in the presence of different metal ions. The data represents the average of 3 independent experiments. C) Representative gel showing products of DNA unwinding. Labeled forked DNA substrate (T1+B1- a 30mer complementary region with non-complementary 30mer tails) was incubated with 2000 nM Mtb XPB in the presence of 2 mM Mg^2+^ and 2 mM of each NTP or dNTP for 30 min at 37°C. HD – heat-denatured DNA substrate. The control reaction lacked nucleotide (-). D) Quantitation of Mtb XPB unwinding activity in the presence of different nucleotides. Data shown are the average of three independent experiments and standard deviation (error bars).

**Figure 5:**
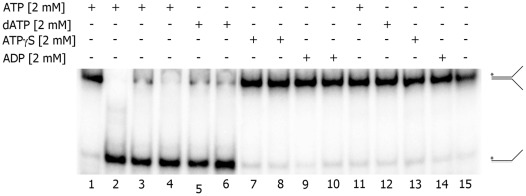
Requirement of ATP hydrolysis for Mtb XPB unwinding activity. Labeled forked DNA substrate was incubated with Mtb XPB in the presence of 2 mM ATP, dATP, ATPγS, or ADP for 30 min at 37°C. Lane 1. no enzyme; lane 2. heat-denatured substrate; Lanes 3, 5, 7 and 9. 1000 nM Mtb XPB; Lanes 4, 6, 8 and 10. 2000 nM Mtb XPB; lanes 11–14. ‘mock’ preparation (1∶10 dilution); lane 15. reaction containing Mtb XPB (2000 nM) and without any nucleotide cofactors.

The metal ion and nucleotide requirements for Mtb XPB helicase were determined in reactions containing a forked DNA substrate (as described above). Maximum unwinding activity was observed in the presence of 2 mM ATP and 2 mM Mg^2+^ (Fig. S2). When we replaced the 2 mM Mg^2+^ with 2 mM Mn^2+^, Ca^2+^, Cu^2+^, Co^2+^, Fe^2+^, Ni^2+^, or Zn^2+^ in the reactions containing 2 mM ATP, Mtb XPB was active only in the presence of Mn^2+^ (Fig. 4A). The extent of DNA unwinding was similar in the presence of Mn^2+^ or Mg^2+^ (about 80%) (Fig. 4B). When we tested the nucleotide requirements in the presence of 2 mM Mg^2+^, unwinding activity was observed with ATP (89%) or dATP (70%), but not in the presence of other nucleotides tested (Fig. 4C and 4D). In the absence of either metal ion or nucleotide, we did not observe DNA unwinding activity of Mtb XPB (Fig. 4, A and C). All the negative control experiments were performed in the absence of Mtb XPB, but in the presence of Mg^2+^ and ATP. We further examined the requirement of ATP hydrolysis for the unwinding activity of Mtb XPB. The experiments were done in the presence of ATP, dATP, ATPγS (non-hydrolyzable ATP), or ADP and the results clearly indicated that unwinding activity required ATP hydrolysis (Fig. 5).

#### Mtb XPB requires a 15–20 nucleotide 3′ extension for efficient unwinding

**Figure 6:**
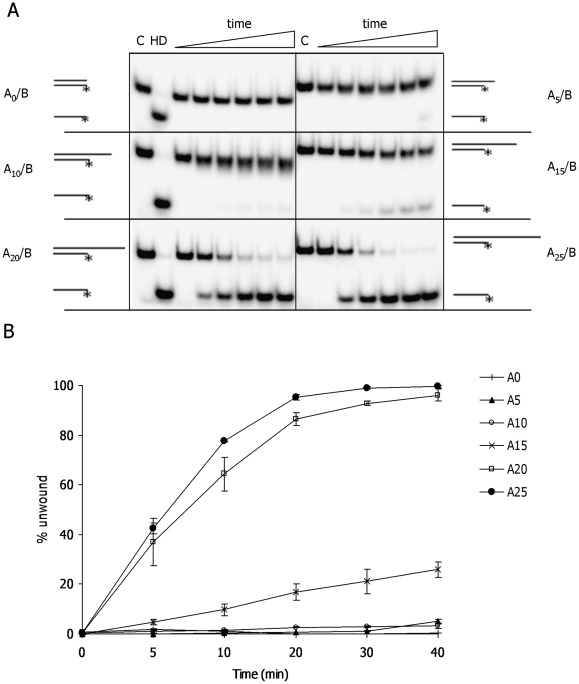
DNA unwinding activity on DNA substrates with 3′ ssDNA overhangs of different length. A) Representative gels of Mtb XPB (2000 nM) unwinding activity on DNA substrates with 0, 5, 10, 15, 20 and 25 nt 3′ –overhangs (A0, A5, A10, A15, A20 and A25). Reactions were incubated for 0, 5, 10, 20, 30 and 40 min. Controls: C-reaction without Mtb XPB incubated for 40 min; HD–heat-denatured substrate. B) The average of 3 independent experiments and standard deviations (error bars) are shown.

Previous studies showed that Mtb XPB helicase catalyzes DNA unwinding in the 3′→5′ direction [10]. Here, we examined the minimal length of 3′ ssDNA required for initiation of Mtb XPB unwinding. For this purpose, DNA unwinding was analyzed using DNA substrates with 20 bp dsDNA and a 0, 5, 10, 15, 20, or 25 nucleotides (nt) 3′ dT overhang (Table S1). The results showed that Mtb XPB did not unwind blunt end dsDNA and very inefficiently unwound DNA substrates with 5 and 10 nt overhangs (<5% input DNA unwound) and partially unwound DNA substrates with a 15 nt 3′ overhang (Fig. 6A). For optimal unwinding under the chosen experimental conditions, Mtb XPB required a 3′ overhang of 20 or 25 nt in length (Fig. 6).

### Mtb XPB exhibited unwinding activity on DNA replication and recombination intermediates

**Figure 7:**
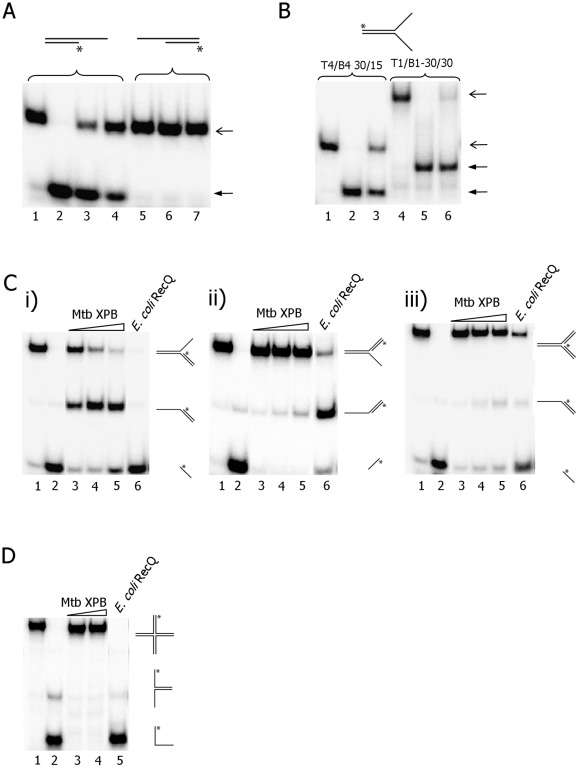
Mtb XPB unwinding of DNA substrates that represent DNA replication and recombination intermediates. The oligos used in this experiment are given in Table S1 and S2. A) 3′ and 5′ overhangs. Lanes 1 and 5. no enzyme; lane 2. heat-denatured DNA substrate; lanes 3 and 6. 2000 nM Mtb XPB; lanes 4 and 7. 1000 nM Mtb XPB. Open arrow- dsDNA; closed arrow- unwound products. B) forked DNA substrates comprising 30mer complementary region and 15mer (lanes 1–3) or 30mer (lanes 4–6) non-complementary tail. Lanes 1 and 4. substrates alone; lanes 2 and 5. heat-denatured substrates; lanes 3 and 6. 2000 nM Mtb XPB. C) i) 3′ flap; ii) 5′ flap; iii) 3 way junction. Lane 1. no enzyme; lane 2. heat denatured substrate; lanes 3–5. increasing concentration of XPB Mtb, 500 nM, 1000 nM and 2000 nM, respectively; lane 6. 10 nM *E. coli* RecQ. D) Holliday junction. Lane 1. substrate alone; lane 2. heat-denatured substrate; lanes 3–4. 1000 nM and 2000 nM Mtb XPB, respectively; lanes 5. 10 nM *E. coli* RecQ.

Mtb XPB helicase was challenged with additional DNA structures, including 3′- and 5′-overhangs, forked, 3′- and 5′-flaps, a nicked 3-way junction and a Holliday junction (Table S2), which resemble intermediates in DNA replication or DNA recombination. As previously reported by Biswas *et* *al*. [10], Mtb XPB was active on a 3′ overhang but not on a 5′ overhang and efficiently unwound forked DNA substrates with up to 30 nt arms (Fig. 7A and 7B). We also observed that Mtb XPB readily unwound 3′ flap DNA but not 5′ flap DNA (Fig. 7C, i and ii) [10]. Since flap structures resemble replication intermediates, this observation may suggest that Mtb XPB could potentially translocate along the leading DNA strand and unwind dsDNA ahead of a stalled replication fork. Finally, Mtb XPB was challenged with a three-way junction, which resembles a replication fork with no gap on the leading or lagging DNA strand and a Holliday junction, which is a typical recombination intermediate. As expected, Mtb XPB did not unwind these two DNA substrates, likely because neither has a 3′ ssDNA overhang.

### Mtb XPB displaced the invading strand containing a 3′tail from D-loop but not from R–loop substrates

**Figure 8:**
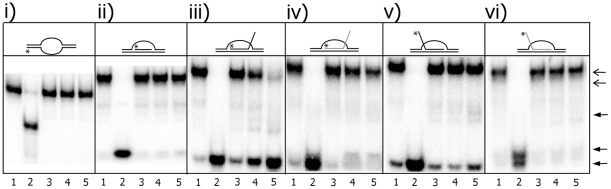
Mtb XPB unwinding activity on bubble, D- and R-loop structures. The oligos used in this experiment are given in Table S1 and S2. i) bubble substrate; ii) D-loop with fully complementary invading strand with no tail; iii) D-loop with 3′ tail; iv) R-loop with 3′ tail; v) D-loop with 5′ tail; vi) R-loop with 5′ tail. Lane 1. no enzyme; lane 2. heat-denatured substrate; lanes 3–5. 500, 1000 and 2000 nM Mtb XPB, respectively. Open arrow- complex DNA substrates; closed arrow- unwound products.

RNA oligonucleotides were made as 2′-OMe modified RNA. This RNA provides a more stable and rigid functional analog of natural RNA [29], [30]. First, we examined the Mtb XPB unwinding activity on DNA:RNA hybrids. For this purpose, DNA oligo D2 was annealed with D3, R1, D4 or R2 (D3 and R1 as well as D4 and R2 were composed of the same nucleotide sequences) (Table S1). Mtb XPB efficiently unwound DNA:DNA or DNA:RNA structures, since the D2 DNA oligo provided a 3′ overhang (Fig. S3). Mtb XPB unwinding activity was then examined using bubble, D- and R-loop substrates. The DNA bubble structure and DNA oligonucleotide fully complementary to one of the unpaired DNA strands in the bubble remained unwound when incubated with Mtb XPB (Fig. 8, i and ii and Table S2). As expected, Mtb XPB was capable of unwinding D-loop structure with a 3′ tail but not with a 5′ tail (Fig. 8, iii and v). However, R-loops with either a 3′ or 5′ non-homologous tail were resistant to unwinding by Mtb XPB (Fig. 8. iv and vi). These results indicated that Mtb XPB required a 3′ DNA tail to exert its unwinding activity, even though the enzyme bound D- and R- loops (Fig. S4).

### Mtb XPB facilitates strand annealing

**Figure 9:**
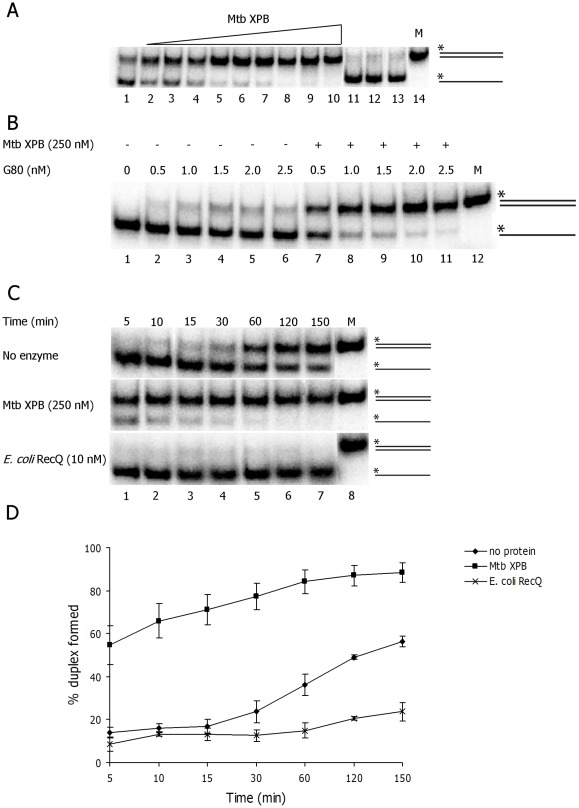
DNA strand annealing activity of Mtb XPB. A) Enzyme concentration-dependent strand annealing activity of Mtb XPB. Labeled C80 oligo (1 nM) incubated with unlabeled G80 oligo (1 nM) in the absence of ATP and increasing concentration of Mtb XPB for 15 min. Lane 1. no enzyme; lanes 2–10. Mtb XPB [nM] 25, 50, 100, 200, 400, 800, 1600, 2000 and 5000 respectively; lanes 11–13. mock dilution: 1∶1, 1∶10, 1∶100, respectively; lane 14. M- duplex marker (80 bp). (B) Unlabeled G80 oligo concentration-dependent strand annealing activity of Mtb XPB. Labeled C80 oligo incubated for 15 min with increasing concentration of unlabeled G80 oligo and with/without 250 nM Mtb XPB in the absence of ATP. Lanes 1–6. reactions in the absence of Mtb XPB (-); lanes 7–11. reactions in the presence of Mtb XPB; lane 11. M- duplex marker (80 bp). C) Time course of strand annealing activity carried out at different time intervals in the absence of enzyme or in the presence of Mtb XPB [250 nM] or *E. coli* RecQ [10 nM]. M- duplex marker (80 bp). D) Quantitation of % reaction product at the indicated time points.

Mtb XPB was also tested for its ability to anneal fully complementary 80 nt DNA oligonucleotides (1 nM each) (Table S1), one of which was labeled with [γ^32^P]ATP. First, the DNA annealing assay was performed with increasing concentrations of Mtb XPB. The efficiency of strand annealing increased with increasing Mtb XPB concentration (Fig. 9A). No strand annealing activity was observed with volume titration of ‘mock’ preparation (Fig. 9A, lanes 11–13). The DNA annealing was also performed with increasing concentration of unlabeled oligo G80 (0.5–2.5 nM) with/without Mtb XPB in the reactions. Efficient strand annealing was clearly seen with increasing concentration of G80 oligo in the presence of Mtb XPB while no annealing was seen in the absence of enzyme (Fig. 9B). Spontaneous annealing of DNA oligos was minimal (about 30%) after 60 min and reached ≈ 50% after 150 min (Fig. 9C and 9D), whereas Mtb XPB catalyzed 66% strand annealing in 10 min (Fig. 9C and 9D). In contrast, considerable strand annealing was not catalyzed by *E. coli* RecQ, Mtb SSB or *E. coli* UvrD under same experimental conditions (Fig. 9C and S5). The presence of Mtb SSB (10 nM) in the reactions as well as incubation of reaction mixtures on ice before incubating at 37°C inhibited the strand annealing activity of Mtb XPB to some extent (Fig. S6). We also tested Mtb XPB unwinding activity on forked substrate (T1+B1) in the absence of unlabeled competitor or in the presence of 10 nM Mtb SSB (Fig. S7). While a reduced unwinding was observed in the absence of unlabeled competitor (due to re-annealing) the presence of Mtb SSB inhibited the unwinding activity. These experiments suggest that Mtb XPB has intrinsic DNA strand annealing activity.

**Figure 10:**
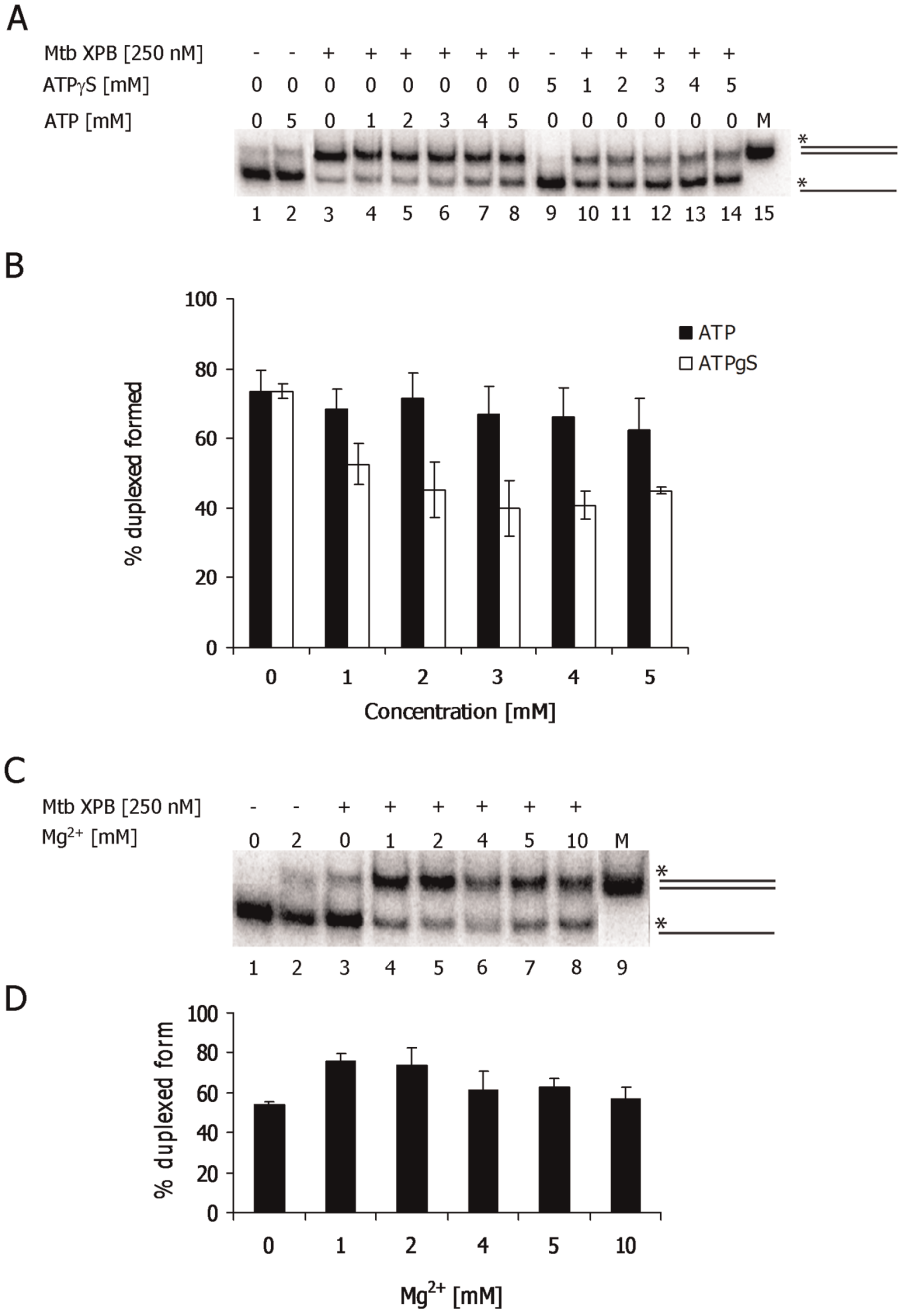
ATP and Mg^2+^-dependence of DNA Mtb XPB strand annealing. A) Strand annealing assays were performed in the presence of 250 nM Mtb XPB with increasing concentration of ATP (lanes 4–8) or ATPγS (lanes 10–14) and 2 mM Mg^2+^. Controls, lane 1. no enzyme and nucleotides; lane 2, without enzyme and with 5 mM ATP; lane 9. without enzyme and with 5 mM ATP-analogue; lane 15. M-duplex marker (80 bp). B) The average of 3 independent experiments and standard deviations (error bars) are shown. C) Strand annealing activity of Mtb XPB (250 nM) in the presence of increasing concentration of Mg^2+^ in the absence of ATP. Lane 1. control reaction without enzyme and Mg^2+^; lane 2. without enzyme and with 2 mM Mg^2+^; lane 3–8. increasing [Mg^2+^] from 0–10 mM, as indicated; lane 9. M- duplex marker (80 bp). D) Reactions were performed in triplicates and average % product calculated. Error bars show standard deviation from the average value.

We further investigated the effect of ATP and Mg^2+^ concentration on the DNA strand annealing activity of Mtb XPB. Mtb XPB catalyzed DNA strand annealing efficiently in the absence or presence of moderate concentrations of ATP (Fig. 10A, lanes 4–8), but the DNA annealing reaction was slightly inhibited by increasing concentrations of ATP or non-hydrolysable ATPγS (Fig. 10B). These results suggest that ATP hydrolysis is not required for optimal strand annealing, but that Mtb XPB may be competent for nucleotide binding during the strand annealing reaction. In contrast, Mg^2+^ is required for Mtb XPB-mediated DNA strand annealing in the presence or absence of ATP (Fig. 10C), although Mn^2+^ or Ca^2+^ also supported the reaction (data not shown). In the absence of ATP, optimal strand annealing was observed in the presence of 1–2 mM Mg^2+^ (Fig. 10D).

## Discussion

Mtb XPB is an ATP-dependent 3′→5′ DNA helicase with DNA-dependent ATPase activity [10]. Human XPB and XPD helicases are subunits of TFIIH, a protein complex that plays essential roles in nucleotide excision repair and RNA polymerase II transcription initiation. However, bacterial XPB is not thought to participate in a TFIIH-like protein complex formation since homologs of other subunits of the TFIIH complex have not yet been identified in bacteria, and the exact biological role(s) of bacterial XPB and XPD are not well understood. Therefore, the goal of this study was to characterize Mtb XPB catalytic activities and DNA substrate specificity *in vitro*, thus enhancing our understanding of its role in DNA repair or other cellular functions.

Comparison of the protein sequences of XPB homologs across multiple phyla revealed that archaeal, bacterial and eukaryotic XPB contain two RecA-like domains within the helicase core region. The *A. fulgidus* XPB 3D structure, which is the only available structure to date, was used to compare the structural organization of XPB from other organisms. The helicase core of Mtb XPB includes the Q-motif [31], seven classical helicase motifs [27] as well as an absolutely conserved XPB-specific motif (RED) [26] (Fig. 1E). The thumb motif (ThM) found as an insertion in the C-terminal RecA-like domain in *A. fulgidus* XPB [26] is much shortened, most likely completely missing, in Mtb XPB as well as in homologs from bacteria and eukaryotes (Fig. 1E). In eukaryotic TFIIH, the RED motif of XPB stimulates the DNA-dependent ATPase activity and is thereby thought to anchor TFIIH to DNA [32].

In addition to the helicase core, eukaryotic and bacterial XPB homologs contain a unique N-terminal domain that appears to be absent in *A. fulgidus* XPB. Although it was not possible to model the 3D structure of the N-terminal XPB domain, there are several conserved amino acids of interest within the N-terminal domain. Within this domain, a F99S substitution in human XPB found in XP patients [16] weakened the interaction between TFIIH p52 and XPB, and resulted in reduced ATPase activity [13]. Phe99 in human XPB is conserved in yeast and other eukaryotes, but not in bacteria or archaea (Fig. 1D). A putative helix-turn-helix DNA binding domain was also predicted in this N-terminal domain [8]. This indicates that the N-terminal domain of Mtb XPB might play a crucial role in DNA-protein and/or protein-protein interactions. In the context of the TFIIH complex, the ATPase activity of XPB is required for DNA unwinding during NER and RNA transcription, while its helicase activity is required for promoter escape during RNA transcription [33]. The sequence and domain identities between eukaryotic and bacterial XPB suggest possible functional identity or overlap, even though it is likely that bacterial XPB acts independent of a TFIIH-like complex. *A. fulgidus* is apparently a slightly atypical XPB since it has a DRD and ThM that is lacking in most XPBs and it appears not to have a standard XPB N-terminal domain. Mtb XPB might be a useful model system for studying eukaryotic XPB and elucidating the structure-function relationships of the N-terminal domain, since it has all the same domains as human XPB, but none of the structurally disordered segments found in human XPB.

The present study demonstrated that Mtb XPB bound ssDNA and dsDNA substrates with variable affinity. Even though Mtb XPB bound these substrates, it did not unwind blunt duplex DNA and bubble DNA. In the band-shift analysis, we observed slowly migrating bands when longer oligos (>60mer) were incubated with high concentrations of Mtb XPB (Fig. 2). This phenomenon may be due to either a high protein:DNA ratio or protein binding to both nonspecific and specific sites [34]. The binding of ssDNA and dsDNA to Mtb XPB suggests that the enzyme may utilize these properties for its unwinding and annealing activities. Even though clear homologs of other subunits of the TFIIH complex, except XPB and XPD, have not been found in bacteria, existence of other proteins that are acting together with XPB can not be excluded. It has recently been reported that in archaea, XPB forms a complex with Bax1 (Binds archaeal XPB) and functions as a helicase-nuclease complex [35]. Existence of such a complex of XPB in bacteria has not been identified yet. The present study also demonstrated that Mtb XPB helicase was active *in vitro* only when present in high molar excess with respect to its DNA substrate. Recently, Biswas *et* *al*. reported similarly that the ATPase activity of XPB is relatively inefficient (50 ATP hydrolyzed per minute per monomer of XPB) [10]. Generally, *M. tuberculosis* helicases exerted their activities at concentrations >100 nM [10], [23]. This might be due to the metabolic conditions inside an intracellular pathogen such as *M. tuberculosis,* which might be considerably different to those of *E. coli* cells. One could also speculate that more enzyme is needed to carry out DNA unwinding processes, since the *M. tuberculosis* genome is particularly rich in GC content (66%) and repeat sequences, in addition to its long replication time [36].

Mtb XPB was equally active in the presence of Mn^2+^ and Mg^2+^ (Fig. 4B), but was inactive in the presence of other metal ion cofactors tested. This observation corroborated the reported cation requirement for ATPase activity of Mtb XPB [10]. In addition, in the presence of Ca^2+^, a lower rate of Mtb XPB ATPase activity was seen previously [10], but unwinding activity was not observed in the presence of Ca^2+^ in our study. In contrast, Mtb UvrD helicase is active in the presence of Mg^2+^, Mn^2+^, Cu^2+^, Co^2+^ or Ni^2+^ [23]. Cockayne syndrome protein (CSB) ATPase is activated by Ca^2+^ [37], while Werner syndrome (WRN) helicase is active in the presence of Mg^2+^, Mn^2+^ or Ni^2+^ (44). All these observations indicate that the metal ion dependent catalytic activity differs considerably among helicases. Mtb XPB helicase required ATP or dATP as cofactor, a characteristic shared by Mtb UvrD and *E. coli* Rep and UvrD [23], [38].

Mtb XPB helicase efficiently unwinds DNA substrates containing a ≥20 nt 3′ overhang and partially unwinds DNA substrates with a 15 nt 3′ overhang. This finding indicates that 15 to 20 nt ssDNA is required for loading Mtb XPB onto DNA and initiation of DNA unwinding. Longer 3′ overhangs may increase the efficiency of unwinding by allowing more than one helicase monomer to bind to each DNA substrate molecule [39]. As expected, Mtb XPB cannot unwind DNA substrates that lack a 3′ ssDNA tail, including blunt-end, bubble, 5′ flap, 3-way junction and Holliday junction DNA substrates. Interestingly, Mtb XPB displaces an incoming/invading DNA strand with a 3′ tail from a D-loop, but does not displace RNA with 3′ tail from an R-loop. These observations clearly indicate that Mtb XPB loads and translocates only on DNA strands containing a 3′ tail. Unwinding of lagging strand replication fork (3′ flap) and D- loop DNA with 3′ extension indicate that Mtb XPB could be active during DNA replication and recombination. However, another prokaryotic helicase, *E. coli* DinG, can unwind D-loops and R-loops [40].

In addition to thoroughly characterizing its unwinding activity, we report for the first time that Mtb XPB efficiently anneals complementary DNA strands in an ATP hydrolysis independent manner. To our knowledge, similar studies for other prokaryotic helicases have not been reported. However, eukaryotic RecQ family helicases WRN, BLM, RecQ1, RecQ5b and DmBLM also have inherent strand annealing activity [41], [42], [43] while *E. coli* RecQ, *E. coli* UvrD and viral NS3 helicases did not show this property [41]. The ability of Mtb XPB to unwind D-loops and anneal complementary strands implicates that this enzyme might be involved in other DNA repair pathways apart from NER. A previously proposed model for synthesis-dependent strand annealing (SDSA) during double strand break repair (DSBR) is consistent with the idea that a single enzyme catalyzes DNA unwinding and DNA annealing. In the SDSA model, the newly synthesized DNA strands are displaced from the template and returned to the broken molecule, allowing the two newly synthesized strands to anneal to each other [44]. Gupta *et al*. [45] recently reported that DSBR in mycobacteria is facilitated by homologous recombination, non-homologous end-joining or single-strand annealing (SSA) pathways while in mammalian and yeast cells, DSBR can also be achieved through an SDSA-like pathway. The SSA pathway occurs only for double-strand breaks flanked by copies of directly duplicated sequence [46]. In this pathway, after resection of one strand on each side of the break, exposing complementary sequences are annealed, followed by flap resection and ligation. Thus, it is possible that an SDSA-like mechanism, in addition to SSA, also promotes DSBR in mycobacteria, and that XPB may play a role in these pathways. However, further genetic studies are necessary to delineate these mechanisms.

In conclusion, we characterized the enzymatic activity and substrate specificity of helicase XPB from Mtb in order to delineate its role in DNA repair and genome maintenance. The studies presented here showed that in addition to its unwinding activity, Mtb XPB possesses intrinsic strand annealing activity. These studies have implications for subsequent delineation of the role of XPB in *M. tuberculosis* genome dynamics and, in turn, on antigenicity and drug resistance development. Clearly, further studies are required to understand the biological significance of the strand annealing and DNA helicase activities of Mtb XPB.

## Materials and Methods

### Structure and sequence analysis

Sequence data was extracted from the NCBI protein sequence databases [47]. Structural disorder predictions were performed with DISOPRED2 [48] and the VSL1 algorithm [49] and secondary structure predictions with PSIPRED v3.0 [50]. The structural models of the Mtb XPB helicase domains were generated from the 2FWR template of the *A. fulgidus* XPB structure [26] employing standard homology modeling with SwissModel [51]. Cofactors were modeled into the XPB structure by structurally aligning the model with the *Thermotoga* RecG structure [52] (structure identifier 1GM5). Multiple sequence alignments were generated with MAFFT [53] from a set of 37 XPB homologs from all three domains of life. The alignments were manipulated in JalView [54], and the protein structure illustrations were generated with PyMOL [55].

### Cloning, expression and purification

Genes encoding *XPB* and single strand binding protein (*ssb*) from *M. tuberculosis* H37Rv (ATCC 25618) and *recQ* from *E. coli* K12 [56], [57] were PCR amplified from respective genomic DNA using primer pairs given in Table S1. The *Mtb XPB, Mtb ssb* and *E. coli recQ* genes were cloned in pET-28b(+) vector. The *Mtb XPB* and *Mtb ssb* were cloned as C-terminal His-tag and *E. coli recQ* cloned as N-terminal His-tag containing proteins. The sequences of the constructs were verified at the Oslo University Hospital, Rikshospitalet DNA sequencing core. The recombinant proteins were expressed in *E. coli* BL21 (DE3) (Mtb XPB) or in ER2566 (Mtb SSB and *E. coli* RecQ). The bacterial cells were grown at 37°C until OD_600_ nm ≈ 0.5 and induced with 0.5 mM IPTG, grown at 18°C overnight, harvested by centrifugation, and frozen at −80°C.

For Mtb XPB, the cell pellet was resuspended in a buffer containing 50 mM NaH_2_PO_4_, pH 8, 600 mM NaCl, 10% Glycerol, 5 mM β-mercaptoethanol, 2 mM MgCl_2_ and Complete protease inhibitor without EDTA (Roche). The cells were disrupted by sonication and the lysate was treated with 2.5 U/ml benzonase (Novagen) to remove nucleic acid contaminants. The lysate was cleared by centrifugation, imidazole added to 10 mM final concentration and the protein was loaded onto a Ni-NTA column (Qiagen). The column was washed and the protein eluted with increasing concentrations of imidazole up to 250 mM in the same buffer but without MgCl_2_. The fractions containing Mtb XPB were concentrated by ultrafiltration (Amicon) and injected onto a Superdex 200 size exclusion column (GE Healthcare) equilibrated and run with a buffer containing 40 mM Tris-HCl, pH 8, 600 mM NaCl, 10% glycerol and 1 mM DTT. The fractions containing the purest protein were pooled and used in the biochemical assays (Figure S1). A mock preparation from the host strain harboring vector without insert was also carried out with the same procedure but without the Superdex 200 step and the eluates were dialyzed immediately against buffer containing 40 mM Tris-HCl, pH 8, 600 mM NaCl, 10% glycerol and 1 mM DTT. The mock preparation was used in all assays to verify any cell contamination that might contribute to the activity.

For *E. coli* RecQ, the cell pellet was resuspended in buffer containing 50 mM NaH_2_PO_4_, pH 8, 300 mM NaCl, 10 mM β-mercaptoethanol and Complete protease inhibitor without EDTA (Roche). The cells were disrupted by sonication and the lysate was treated with 6.25 U/ml benzonase to remove nucleic acid contaminants. To the cleared lysate, 10 mM imidazole was added before purifying the protein on a Ni-NTA column according to the manufacturer's directions (Qiagen, Germany). The eluted recombinant protein was dialyzed immediately against a buffer containing 50 mM NaH_2_PO_4_, pH 8.0, 300 mM NaCl and 10 mM β-mercaptoethanol. The His-tag was cleaved off by adding thrombin (Sigma-Aldrich) at 1∶500 wt:wt ratio, and incubated on ice for 14 hours. The cleaved protein, after addition of 10% glycerol to final concentration, was kept at −80°C.

For Mtb SSB, the cell pellet was resuspended in a buffer containing 50 mM NaH_2_PO_4_, pH 8, 300 mM NaCl, 5 mM β-mercaptoethanol and 10 mM imidazole. The cells were disrupted by sonication, and the protein was purified from the cleared cell lysate on a Ni-NTA column according to the manufacturer's directions (Qiagen). The eluted recombinant protein was dialyzed immediately against a buffer containing 50 mM NaH_2_PO_4_, pH 8.0 and 300 mM NaCl and glycerol was added to 20% before freezing at −80°C.

The identity of the purified proteins was verified by mass spectrometry analysis. Any possible DNA contamination from *E. coli* host cells was verified by running the purified proteins in an agarose gel followed by ethidium bromide staining.

### Preparation of DNA substrates for assays

Desalted DNA oligomers (Table S1) were purchased from Operon Biotechnologies, Inc. Oligonucleotides were 5′-end-labeled using [γ^32^P]ATP and T4 polynucleotide kinase (New England Biolabs) as per the manufacturer's directions. After removal of unincorporated radio-nucleotides by a illustra Microspin™ G-25 column (GE Healthcare, UK), radiolabeled oligo was annealed to complementary oligo at a 1∶2 molar ratio (in the buffer containing 40 mM Tris HCl pH 8.0 and 50 mM NaCl). The reaction mix was heated to 95°C for 5 min and then allowed to cool down slowly to room temperature. For purification of the individual fragment, the labeled fragments were separated on 8% native polyacrylamide gel. The gel pieces containing the required fragments were excised, and DNA was eluted into buffer containing 10 mM Tris-HCl pH 8.0 and 0.5 mM EDTA by incubating overnight at 4°C. The molar concentrations of the substrates were calculated after measuring the specific activity on a liquid scintillation counter. The substrates were stored at 4°C.

### Electrophoretic mobility shift assay (EMSA)

EMSA was carried out by adding Mtb XPB (at indicated concentration) in the buffer (10 µl) containing 40 mM Tris-HCl (pH 8), 2.5 mM EDTA, 2 mM MgCl_2_, 100 µg/ml bovine serum albumin (BSA), 6% glycerol, 1 mM DTT and 100 pM of the indicated DNA substrate. After incubating for 15 min on ice, 2 µl of 60% glycerol was added immediately before loading on to a pre-run (30 min) 5% native PAGE gel (29∶1, acrylamide: bisacrylamide). Electrophoresis was done using low ionic strength buffer (6.7 mM Tris HCl pH 8, 3.3 mM sodium-acetate pH 5.5 and 2 mM EDTA pH 8) at 200 V for 5 min followed by 160 V for 85 min in ice water bath with continuous buffer recirculation between the upper and lower chambers. Products were visualized using Typhoon PhosphorImager (Typhoon 9410) and quantitated using ImageQuant software (GE Healthcare). Percent DNA bound was calculated as follows: percent DNA bound  =  (B/(B+F)) ×100, where B is the bound DNA and F is the free DNA.

### Helicase assay

The helicase assay was performed in 10 µl of helicase buffer (20 mM Tris-HCl, pH 7.5, 50 mM NaCl, 1 mM DTT, 2 mM MgCl_2_, 2 mM ATP and 50 µg/ml BSA) using XPB (at indicated concentrations) and 1 nM radiolabeled DNA substrate. The reaction mixtures were immediately incubated at 37°C for 30 min. The reaction was terminated by adding 3x stop dye (50 mM EDTA, 40% glycerol, 0.9% SDS, 0.1% bromophenol blue and 0.1% xylene cyanol) along with 100x molar excess unlabeled competitor DNA (complement of an unlabeled strand) and kept at 37°C for an additional 5 min. The reaction products were analyzed on 6 or 8% native polyacrylamide (19∶1) gels in 1x Tris/ borate/EDTA buffer. Products were visualized using Typhoon PhosphorImager and quantitation was performed using ImageQuant software (GE Healthcare). Percent helicase unwound was calculated as follows: percent unwound  =  (P/(S+P)) ×100, where P is the product and S is the residual substrate. Values of P and S were determined by subtracting background values in controls having no enzyme and heat denatured substrate, respectively.

### DNA strand annealing assay

The DNA strand annealing activity of Mtb XPB was measured using complementary oligonucleotides, one of which was [γ^32^P] 5′ end-labeled as described above. In the strand annealing reactions (10 µl final volume) labeled oligo (1 nM) was added to helicase reaction buffer (20 mM Tris-HCl, pH 8.0, 2 mM MgCl_2_, 40 µg/ml BSA and 1 mM DTT). Where specified, ATP or ATPγS (2 mM) was also added. Subsequently, Mtb XPB, Mtb SSB, *E. coli* RecQ or *E. coli* UvrD (Biohelix, USA) was added at concentrations as specified in the figure legends. Reactions were initiated by the addition of the unlabeled oligonucleotides (1 nM), followed by immediate incubation at 37°C for 15 min. The protein-independent annealing was measured in identical conditions over longer intervals (0–3 h). The reactions were stopped by adding 5 µl 3x stop dye (50 mM EDTA, 40% glycerol, 0.9% SDS, 0.1% bromophenol blue and 0.1% xylene cyanol) along with 10x molar excess of unlabeled competitor oligonucleotide. The reaction products were analyzed on nondenaturing 8% polyacrylamide gels at room temperature in 1x Tris/borate/EDTA. Products were visualized using Typhoon PhosphorImager and quantitated using ImageQuant software.

## Supporting Information

Figure S1 A) Coomassie stained SDS-page gel showing the purification of Mtb XPB on a Ni-NTA column. Lanes 1. Lysate; 2. Pellet; 3. Cleared lysate; 4. Flow-through; 5–7: Wash 10 mM, 20 mM and 30 mM imidazole, respectively; 8–14: Elutions containing 40, 60, 80, 100, 140, 180, 220 mM imidazole, respectively. Elution fractions containing 40–180 mM imidazole were pooled, concentrated and further purified on Superdex 200. B) Coomassie stained SDS-page gel showing the purification of Mtb XPB on a Superdex 200 column. Lanes: 1. Pooled fractions from Ni-NTA; 2–18. fractions from the Superdex 200 column. Fractions in lanes 12–13 were pooled and used in the biochemical assays. Protein molecular weight markers (kD) are indicated on the left.
(TIF)

Figure S2 **Optimization of Mg^2+^ and ATP concentrations needed for unwinding activity of Mtb XPB**. Unwinding activity of Mtb XPB (2000 nM) was titrated with increasing concentrations of Mg^2+^ and ATP using forked DNA substrate (T1+B1). Lanes: 1. no enzyme; 2. heat denatured substrate; 3–8. increasing concentration of ATP in the presence of 2 mM Mg^2+^; 9–14. increasing concentration of Mg^2+^ in the presence of 2 mM ATP.
(TIF)

Figure S3 **Mtb XPB unwinding activity on DNA:RNA hybrid duplexes.** All substrates contain D2 oligonucleotide as bottom strand and annealed with D3, R1, D4 or R2 oligonucleotides. i) DNA:DNA hybrid duplex with D2:D3; ii) DNA:RNA hybrid duplex with D2:R1; iii) DNA:DNA hybrid duplex with D2:D4; iv) DNA:RNA hybrid duplex with D2:R2; Lane 1. substrate alone; lane 2. heat-denatured substrate; lanes 3–5. 500, 1000 and 2000 nM Mtb XPB, respectively. Open arrow- dsDNA substrates; closed arrow- unwound products.
(TIF)

Figure S4 **Mtb XPB binds D- and R- loop substrates.** i) D-loop with 3′ tail (D1+D2+D4); ii) D-loop with 5′ tail (D1+D2+D3); iii) R-loop with 5′ tail (D1+D2+R1); vi) R-loop with 3′ tail (D1+D2+R2). Lanes 1. no enzyme; 2. 2000 nM Mtb XPB.
(TIF)

Figure S5 **Strand annealing activity of Mtb SSB and** ***E. coli*** **UvrD.** Labeled C80 oligo incubated with unlabeled G80 oligo in the absence of ATP and increasing concentration of Mtb SSB or *E. coli* UvrD. Lane 1. no enzyme (-); lanes 2–7. increasing concentration of enzymes 5, 10, 50, 100, 200 and 400 nM, respectively; lane 8. M- duplex marker (80 bp).
(TIF)

Figure S6 **Influence of Mtb SSB and cold incubation on strand annealing activity of Mtb.** A) Labeled C80 oligo incubated with unlabeled G80 oligo, increasing concentrations of Mtb XPB and 10 nM Mtb SSB. Lanes 1–4. reactions in the absence of 10 nM Mtb SSB; lanes 5–8. reactions in the presence of 10 nM Mtb SSB; lane 8. M- duplex marker (80 bp). B) Labeled C80 oligo incubated with unlabeled G80 oligo in the presence of increasing concentrations of Mtb XPB. Lanes 1–4. reactions kept at 37^°^C for 15 min only; lanes 5–8. reactions kept on ice for 15 min and then kept at 37^°^C for 15 min; lane 8. M- duplex marker (80 bp).
(TIF)

Figure S7 **Unwinding activity of Mtb XPB in the absence of unlabeled competitor or in the presence of Mtb SSB**. Unwinding activity of Mtb XPB was titrated with increasing concentration of Mtb XPB in the absence of unlabeled competitor or in the presence of 10 nM Mtb SSB. Lanes: 1. no enzyme; 2. heat denatured substrate; 3–5. in the presence unlabeled competitor (T1); 6–8. in the absence of unlabeled competitor; 9–11 in the presence of 10 nM Mtb SSB; 12. Mtb SSB alone incubated with forked substrate.
(TIF)

Table S1 DNA oligos used in this study.
(PDF)

Table S2 Summary of DNA unwinding activity of Mtb XPB.
(PDF)
