# Supplementary figures and images for: Enzymatic Activities and DNA Substrate Specificity of Mycobacterium tuberculosis DNA Helicase XPB
Source: PLoS One. 2012 May 16;7(5):e36960. doi: 10.1371/journal.pone.0036960 (PMC3353954; doi:10.1371/journal.pone.0036960)

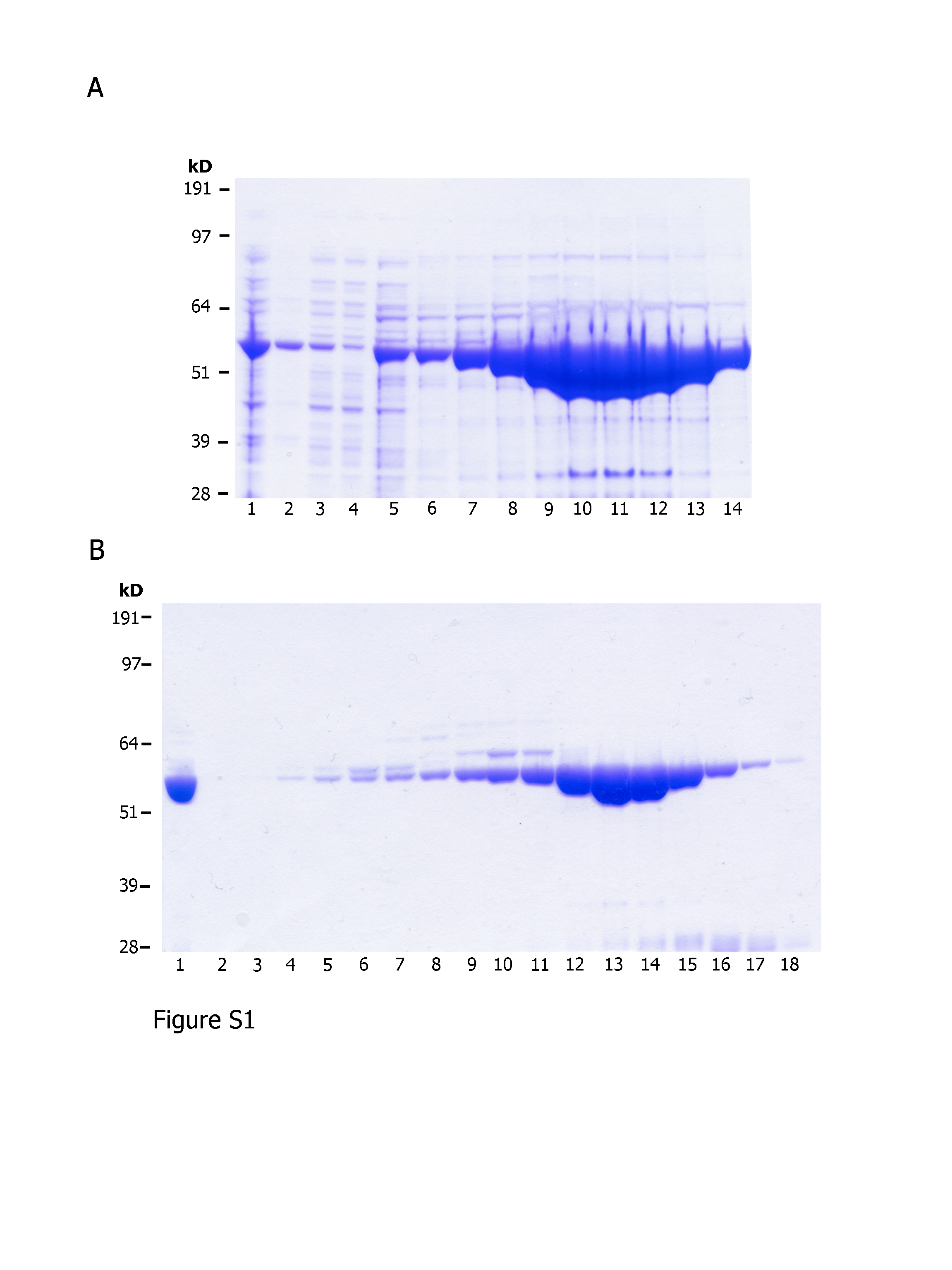

Supplement: Figure S1 — A) Coomassie stained SDS-page gel showing the purification of Mtb XPB on a Ni-NTA column. Lanes 1. Lysate; 2. Pellet; 3. Cleared lysate; 4. Flow-through; 5–7: Wash 10 mM, 20 mM and 30 mM imidazole, respectively; 8–14: Elutions containing 40, 60, 80, 100, 140, 180, 220 mM imidazole, respectively. Elution fractions containing 40–180 mM imidazole were pooled, concentrated and further purified on Superdex 200. B) Coomassie stained SDS-page gel showing the purification of Mtb XPB on a Superdex 200 column. Lanes: 1. Pooled fractions from Ni-NTA; 2–18. fractions from the Superdex 200 column. Fractions in lanes 12–13 were pooled and used in the biochemical assays. Protein molecular weight markers (kD) are indicated on the left. (TIF) [file pone.0036960.s001.tif]

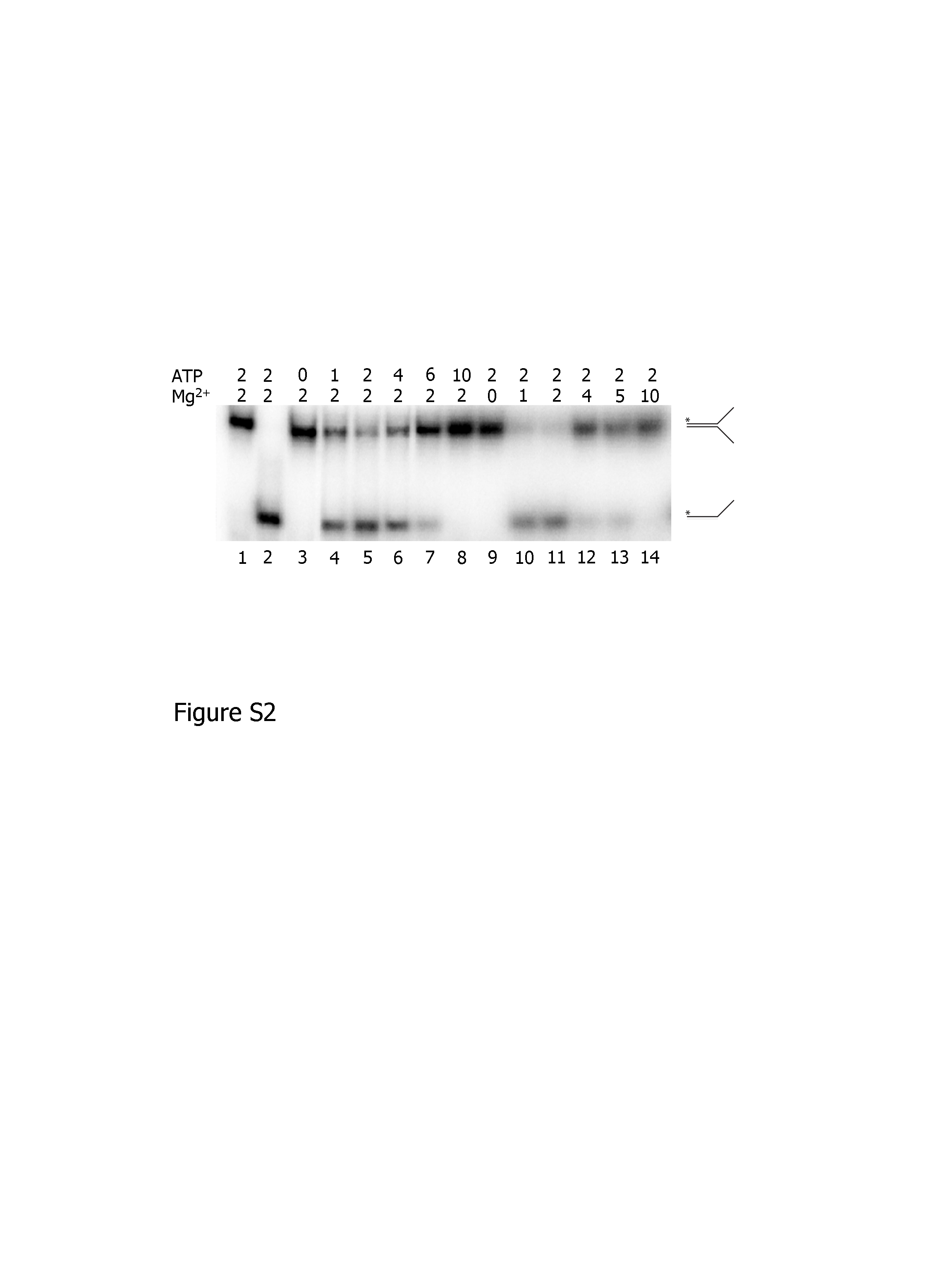

Supplement: Figure S2 — Optimization of Mg2+ and ATP concentrations needed for unwinding activity of Mtb XPB. Unwinding activity of Mtb XPB (2000 nM) was titrated with increasing concentrations of Mg2+ and ATP using forked DNA substrate (T1+B1). Lanes: 1. no enzyme; 2. heat denatured substrate; 3–8. increasing concentration of ATP in the presence of 2 mM Mg2+; 9–14. increasing concentration of Mg2+ in the presence of 2 mM ATP. (TIF) [file pone.0036960.s002.tif]

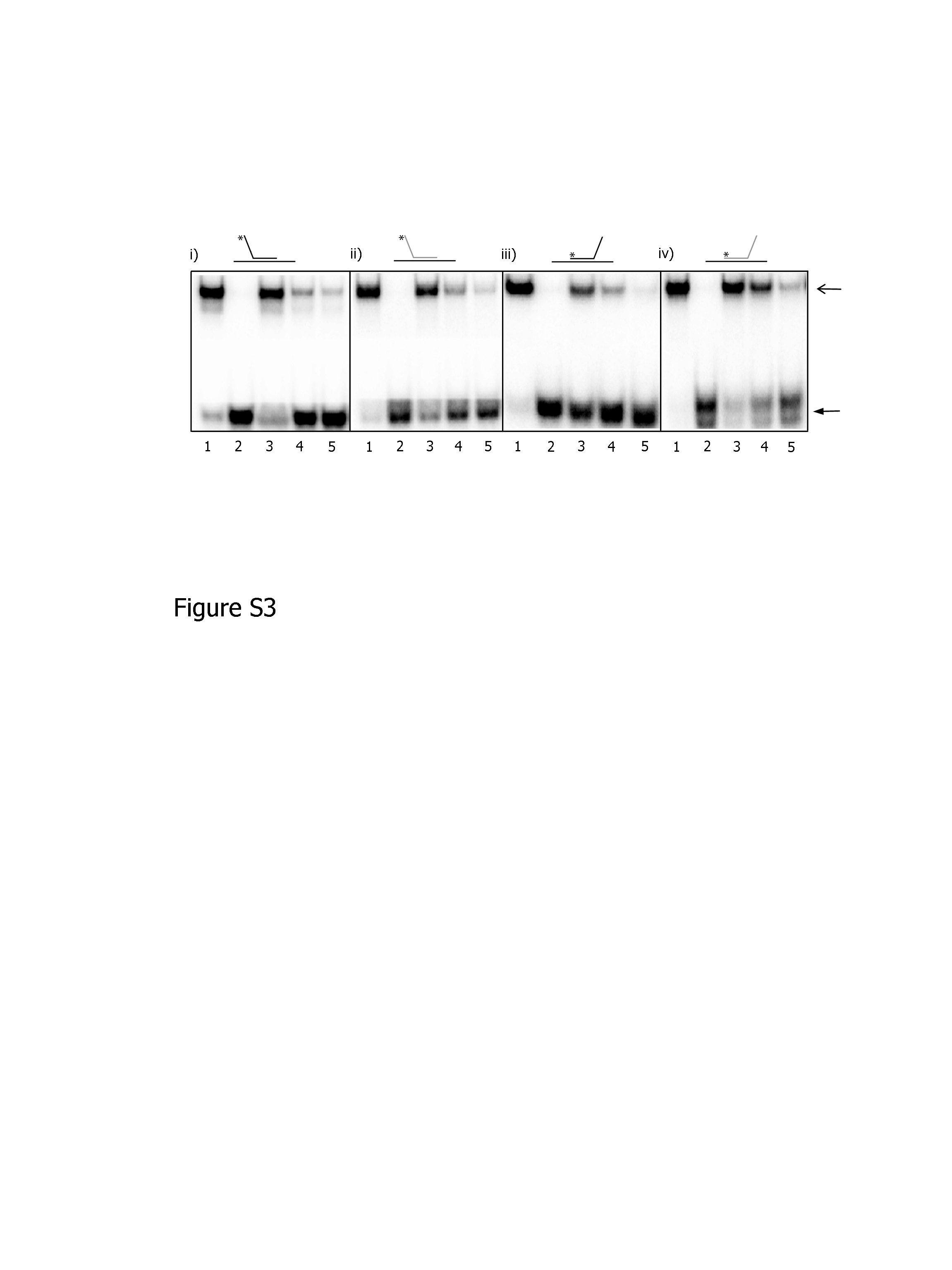

Supplement: Figure S3 — Mtb XPB unwinding activity on DNA:RNA hybrid duplexes. All substrates contain D2 oligonucleotide as bottom strand and annealed with D3, R1, D4 or R2 oligonucleotides. i) DNA:DNA hybrid duplex with D2:D3; ii) DNA:RNA hybrid duplex with D2:R1; iii) DNA:DNA hybrid duplex with D2:D4; iv) DNA:RNA hybrid duplex with D2:R2; Lane 1. substrate alone; lane 2. heat-denatured substrate; lanes 3–5. 500, 1000 and 2000 nM Mtb XPB, respectively. Open arrow- dsDNA substrates; closed arrow- unwound products. (TIF) [file pone.0036960.s003.tif]

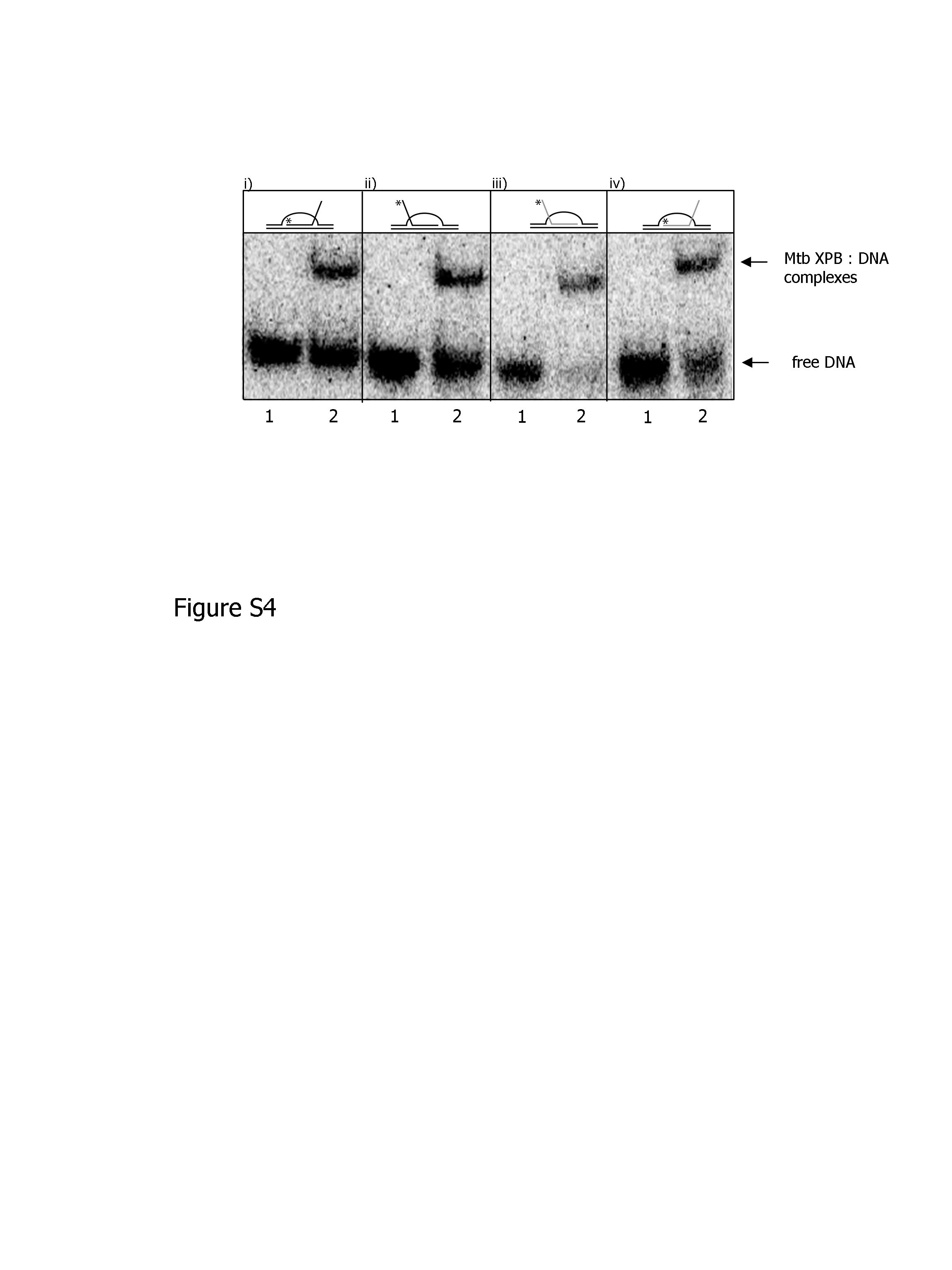

Supplement: Figure S4 — Mtb XPB binds D- and R- loop substrates. i) D-loop with 3′ tail (D1+D2+D4); ii) D-loop with 5′ tail (D1+D2+D3); iii) R-loop with 5′ tail (D1+D2+R1); vi) R-loop with 3′ tail (D1+D2+R2). Lanes 1. no enzyme; 2. 2000 nM Mtb XPB. (TIF) [file pone.0036960.s004.tif]

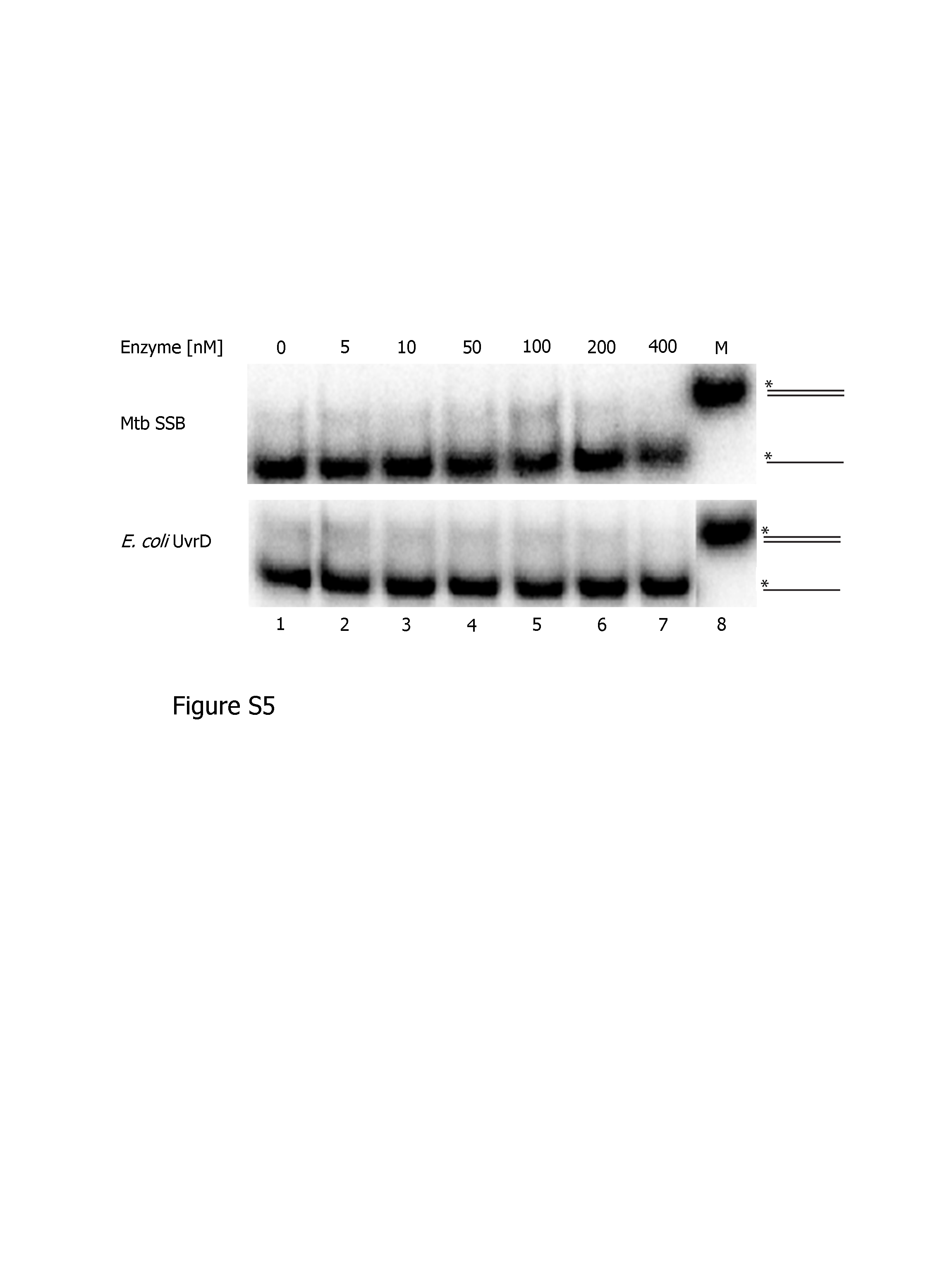

Supplement: Figure S5 — Strand annealing activity of Mtb SSB and E. coli UvrD. Labeled C80 oligo incubated with unlabeled G80 oligo in the absence of ATP and increasing concentration of Mtb SSB or E. coli UvrD. Lane 1. no enzyme (-); lanes 2–7. increasing concentration of enzymes 5, 10, 50, 100, 200 and 400 nM, respectively; lane 8. M- duplex marker (80 bp). (TIF) [file pone.0036960.s005.tif]

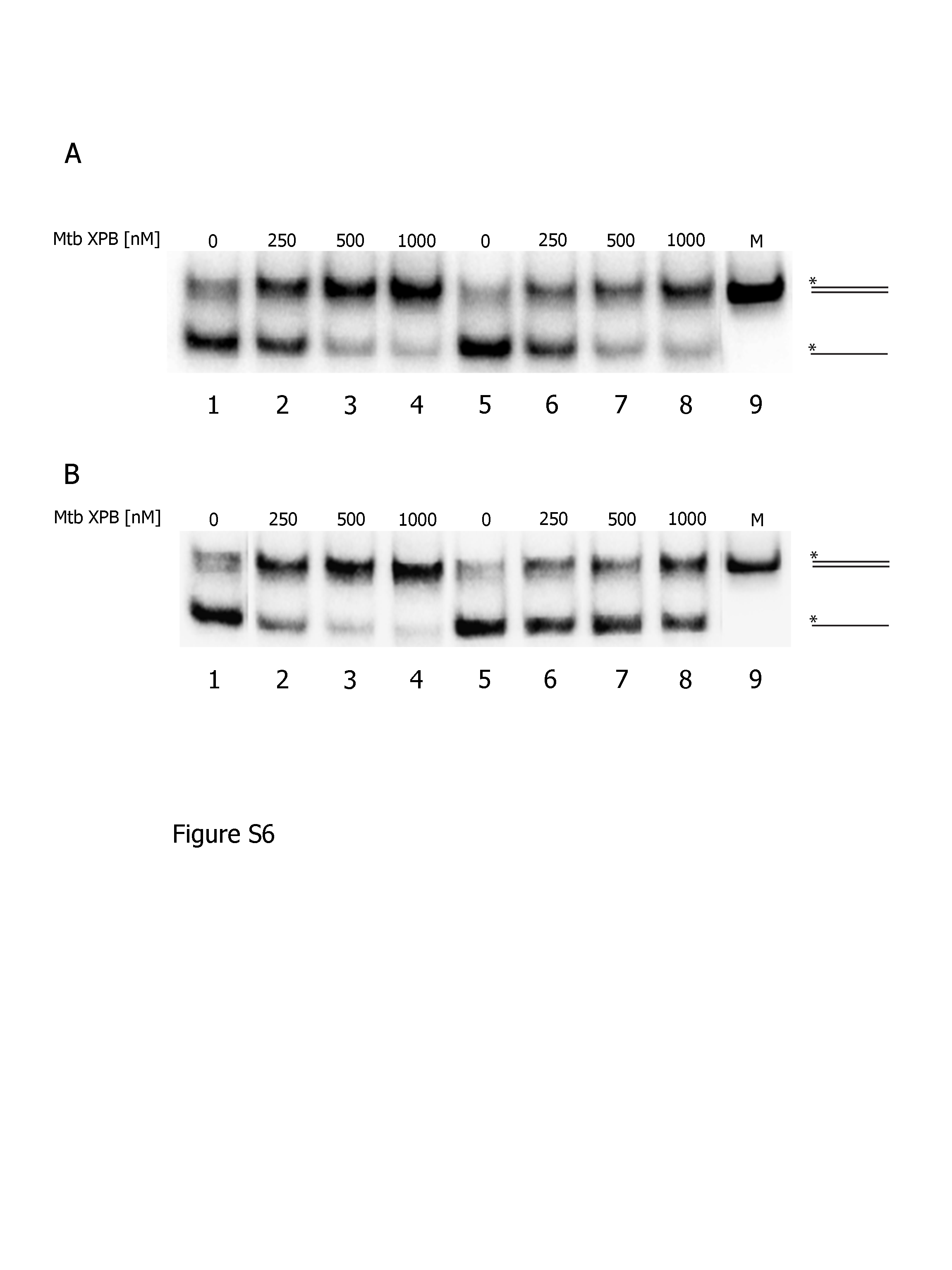

Supplement: Figure S6 — Influence of Mtb SSB and cold incubation on strand annealing activity of Mtb. A) Labeled C80 oligo incubated with unlabeled G80 oligo, increasing concentrations of Mtb XPB and 10 nM Mtb SSB. Lanes 1–4. reactions in the absence of 10 nM Mtb SSB; lanes 5–8. reactions in the presence of 10 nM Mtb SSB; lane 8. M- duplex marker (80 bp). B) Labeled C80 oligo incubated with unlabeled G80 oligo in the presence of increasing concentrations of Mtb XPB. Lanes 1–4. reactions kept at 37°C for 15 min only; lanes 5–8. reactions kept on ice for 15 min and then kept at 37°C for 15 min; lane 8. M- duplex marker (80 bp). (TIF) [file pone.0036960.s006.tif]

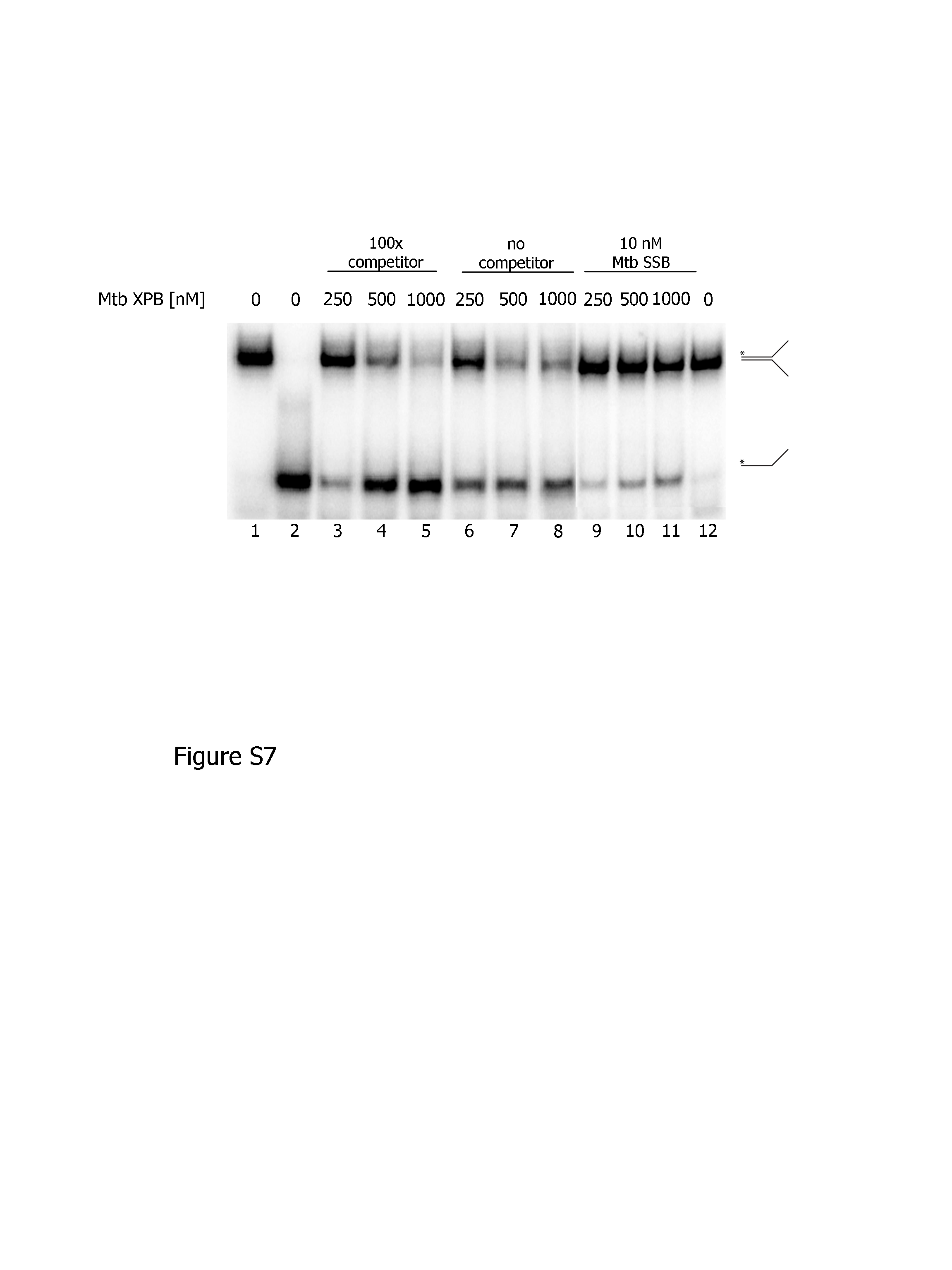

Supplement: Figure S7 — Unwinding activity of Mtb XPB in the absence of unlabeled competitor or in the presence of Mtb SSB. Unwinding activity of Mtb XPB was titrated with increasing concentration of Mtb XPB in the absence of unlabeled competitor or in the presence of 10 nM Mtb SSB. Lanes: 1. no enzyme; 2. heat denatured substrate; 3–5. in the presence unlabeled competitor (T1); 6–8. in the absence of unlabeled competitor; 9–11 in the presence of 10 nM Mtb SSB; 12. Mtb SSB alone incubated with forked substrate. (TIF) [file pone.0036960.s007.tif]
