# Supplementary material for: Enzymatic Activities and DNA Substrate Specificity of Mycobacterium tuberculosis DNA Helicase XPB
Source: PLoS One. 2012 May 16;7(5):e36960. doi: 10.1371/journal.pone.0036960 (PMC3353954; doi:10.1371/journal.pone.0036960)
Supplement: Table S1 — DNA oligos used in this study. (PDF) [file pone.0036960.s008.pdf]

Table S1

DNA oligos used in this study. Restriction sites are underlined.

Oligos	Sequence (5´→ 3´)	Source
**Gene amplification**		
SVB91 (Mtb *XPB*-F)	cgtctagaaataattttgtttaactttaagaaggagatataccatgcagtccgataagacggtgct	This study
SVB92 (Mtb *XPB*-R)	gcctcgagaattgccgggcccagcaggt	This study
Mtb *ssb*-F	cgtctagaaataattttgtttaactttaagaaggagatataccatggctggtgacaccac	This study
Mtb *ssb*-R	cgctcgaggaatggcggttcgtcat	This study
SVB107-F (*E. coli K12 recQ)*	cgaagcttatggcgcaggcggaagtgttg	This study
SVB108-R (*E. coli K12 recQ)*	cgctcgagctactcttcgtcatcgccat	This study
**Assay substrates**		
T4	GACGCTGCCGAATTCTACCAGTGCCTTGCTAGGACATCTTTGCCCA	(1)
B4	CTAGACAGCTCCATGTAGCAAGGCACTGGTAGAATTCGGCAGCGT	(1)
T1	GACGCTGCCGAATTCTACCAGTGCCTTGCTAGGACATCTTTGCCCACCTGCAG GTTCACCC	(1)
B1	ATCGATAGTCGGATCCTCTAGACAGCTCCATGTAGCAAGGCACTGGTAGAATT CGGCAGCGT	(1)
B2	GGGTGAACCTGCAGGTGGGCAAAGATGTCCTAGCAAGGCACTGGTAG AATTCGGCAGCGTC	This study
B3	GGGTGAACCTGCAGGTGGGCCCCACGACGACGATGGAAACCTGGTAG AATTCGGCAGCGTC	This study
B5	TAGCAAGGCACTGGTAGAATTCGGCAGCGT	This study
B6	ACGCTGCCGAATTCTACCAGTGCCTTGCTA	This study
A	CGTGACATGCCGTGACTAGCTTTTTTTTTTTTTTTTTTTT	(2)
B	GCTAGTCACGGCATGTCACG	(2)
C	TTTTTTTTTTTTTTTTTTTTCGTGACATGCCGTGACTAGC	(2)
A0	CGTGACATGCCGTGACTAGC	This study
A5	CGTGACATGCCGTGACTAGCTTTTT	This study
A10	CGTGACATGCCGTGACTAGCTTTTTTTTTT	This study
A15	CGTGACATGCCGTGACTAGCTTTTTTTTTTTTTTT	This study
A25	CGTGACATGCCGTGACTAGCTTTTTTTTTTTTTTTTTTTTTTTTT	This study
RF1	GTCGGATCCTCTAGACAGCTCCATGATCACTGGCACTGGTAGAATTCGGC	(3)
RF2	CAACGTCATAGACGATTACATTGCTACATGGAGCTGTCTAGAGGATCCGA	(3)
RF3	TAGCAATGTAATCGTCTATGACGTT	(3)
RF4	TGCCGAATTCTACCAGTGCCAGTGAT	(3)
HJ1	GACGCTGCCGAATTCTGGCTTGCTAGGACATCTTTGCCCACGTTGACCC	(4)
HJ2	TGGGTCAACGTGGGCAAAGATGTCCTAGCAATGTAATCGTCTATGACGTT	(4)
HJ3	CAACGTCATAGACGATTACATTGCTAGGACATGCTGTCTAGAGACTATCGA	(4)
HJ4	ATCGATAGTCTCTAGACAGCATGTCCTAGCAAGCCAGAATTCGGCAGCGT	(4)
D1	GGGTGAACCTGCAGGTGGGCGGCTGCTCATCGTAGGTTAGTTGGTAGAATTCG GCAGCGTC	(5)

D2	GACGCTGCCGAATTCTACCAGTGCCTTGCTAGGACATCTTTGCCCACCTGCAG GTTCACCC	(5)
D3	TAAGAGCAAGATGTTCTATAAAAGATGTCCTAGCAAGGCAC	(5)
D4	AAAGATGTCCTAGCAAGGCACGATCGACCGGATATCTATGA	(5)
D5	AAAGATGTCCTAGCAAGGCAC	This study
R1	TAAGAGCAAGATGTTCTATAAAAGATGTCCTAGCAAGGCAC	This study
R2	AAAGATGTCCTAGCAAGGCACGATCGACCGGATATCTATGA	This study
C80	GCTGATCAACCCTACATGTGTAGGTAACCCTAACCCTAACCCTAAGGACAACC CTAGTGAAGCTTGTAACCCTAGGAGCT	(6)
G80	AGCTCCTAGGGTTACAAGCTTCACTAGGGTTGTCCTTAGGGTTAGGGTTAGGG TTACCTACACATGTAGGGTTGATCAGC	(6)

References:

1. Rossi, M. L., Ghosh, A. K., Kulikowicz, T., Croteau, D. L., and Bohr, V. A. (2010) *DNA Repair (Amst)* **9**, 796-804
2. Biswas, T., Pero, J. M., Joseph, C. G., and Tsodikov, O. V. (2009) *Biochemistry* **48**, 2839-2848
3. McGlynn, P., and Lloyd, R. G. (2001) *Proc Natl Acad Sci U S A* **98**, 8227-8234
4. Lloyd, R. G., and Sharples, G. J. (1993) *Embo J* **12**, 17-22
5. McGlynn, P., Al-Deib, A. A., Liu, J., Marians, K. J., and Lloyd, R. G. (1997) *J Mol Biol* **270**, 212-221
6. Machwe, A., Xiao, L., Groden, J., Matson, S. W., and Orren, D. K. (2005) *J Biol Chem* **280**, 23397-23407
