# Supplementary material for: Enzymatic Activities and DNA Substrate Specificity of Mycobacterium tuberculosis DNA Helicase XPB
Source: PLoS One. 2012 May 16;7(5):e36960. doi: 10.1371/journal.pone.0036960 (PMC3353954; doi:10.1371/journal.pone.0036960)
Supplement: Table S2 — Summary of DNA unwinding activity of Mtb XPB. (PDF) [file pone.0036960.s009.pdf]

Table S2. Summary of DNA unwinding activity of Mtb XPB.

**Substrate name**	**Oligonucleotide combination**	**Structure**	**Unwinding activity**
Blunt end duplex	A_0+B*	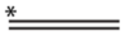	-
3´-overhang	A+B*	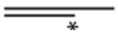	+
5´-overhang	C+B*	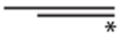	-
Fork	T4*+B4/T1*+B1	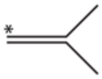	+
3´ flap	RF1+RF2+RF3*	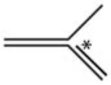	+
5´ flap	RF1+RF2+RF4*	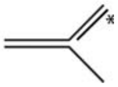	-
Nicked 3-way junction	RF1+RF2+RF3*+RF4	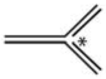	-
Holliday junction	HJ1*+HJ2+HJ3+HJ4	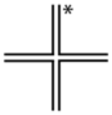	-
Bubble	D1+D2*	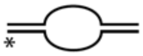	-
D-loop with fully complementary invading strand	D1+D2+D5*	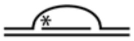	-
3´-tailed D-loop	D1+D2+D4*	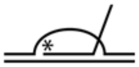	+
3´-tailed R-loop	D1+D2+R2*	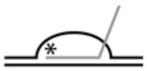	-
5´-tailed D-loop	D1+D2+D3*	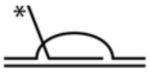	-
5´-tailed R-loop	D1+D2+R1*	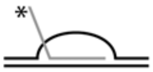	-

* -indicates the 5´-end-labeled substrates
